# Complete Genome Sequences of *Arthrobacter* Phages Eraser, Kaylissa, and Phives

**DOI:** 10.1128/mra.00178-22

**Published:** 2022-04-07

**Authors:** Srinidhi Gadula, Nayan Pallothu, Rodrigue Jean-Baptiste, Joseph Holloway, Leonidas Salichos, Bryan Gibb

**Affiliations:** a Department of Biological and Chemical Sciences, New York Institute of Technology, Old Westbury, New York, USA; Portland State University

## Abstract

Bacteriophages Phives, Kaylissa, and Eraser are siphoviruses infecting Arthrobacter globiformis B-2880 that were isolated in fall 2019 in Long Island, New York, from soil samples collected in Old Westbury, New York. All three bacteriophages are assigned to phage cluster AZ based on gene content similarity. While many aspects of the genomes are similar across the three phages, the endolysin genes for the phages are different and are located in different locations within the genomes.

## ANNOUNCEMENT

**FIG 1:**
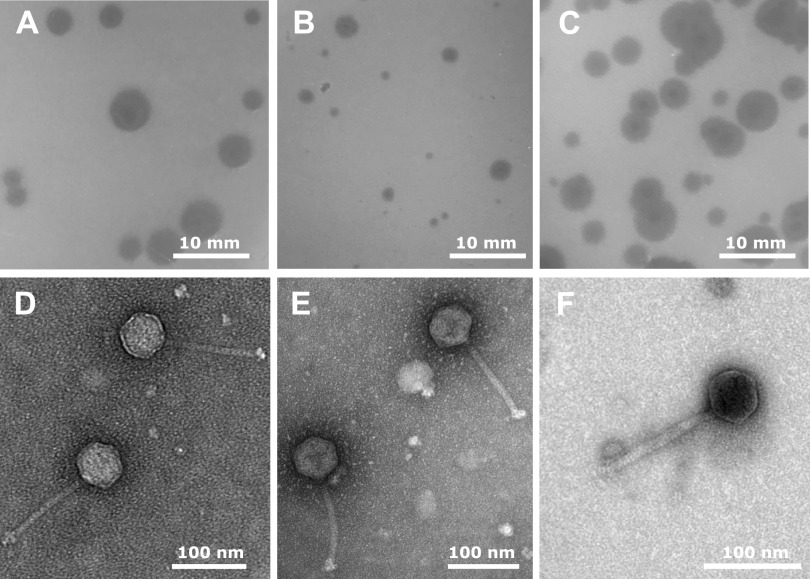
Plaque morphology (A to C) and TEM images (D to F) of *Arthrobacter* phages Eraser (A and D), Phives (B and E), and Kaylissa (C and F). Phages were incubated at 30°C for 48 h prior to imaging. Phage lysates were negatively stained with 1% uranyl acetate for TEM.

Bacteriophages are the most abundant organisms in the biosphere (1). Recent efforts to isolate and characterize bacteriophages are providing valuable insights into host-pathogen evolutionary relationships and uncovering potential therapeutic and biotechnical applications (2–4). *Arthrobacter* phages Phives, Kaylissa, and Eraser were isolated from soil samples from the campus of the New York Institute of Technology in Old Westbury, New York (Table 1), as part of the Science Education Alliance-Phage Hunters Advancing Genomics and Evolutionary Science (SEA-PHAGES) program (5). Phage isolation, plaque purification, and genome extraction were performed according to protocols described in the SEA-PHAGES Discovery Guide (https://seaphagesphagediscoveryguide.helpdocsonline.com/home). All three bacteriophages were isolated by enrichment on Arthrobacter globiformis B-2880 with peptone-yeast-calcium (PYCa) medium at 30°C and purified with three rounds of plaque purification. Plaques of Phives and Kaylissa have a bullseye morphology, while Eraser has larger (8- to 12-mm) round, clear plaques. Negative-stain transmission electron microscopy (TEM) analysis shows that all three phages have icosahedral heads and noncontractile tails (Fig. 1), reflecting siphoviral morphology (6).

**TABLE 1:** Phage GenBank and SRA accession numbers and genome assembly results

Phage	GenBank accession no.	SRA accession no.	Sampling location coordinates	Cluster	Avg coverage (×)	No. of reads (thousands)	Genome size (bp)	GC content (%)	Genome end (3′ overhang)	No. of genes
Phives	MT889376	SRX12198771	40.813139N, 73.604667W	AZ	2,651	775.7	44,204	67.3	CGAAGGGGCAT	70
Kaylissa	MZ005682.1	SRX12198769	40.7887N, 73.5996W	AZ	2,171	669.9	44,124	67.6	CGAAGGGGCAT	71
Eraser	MZ747516	SRX12198766	40.81255N, 73.604333W	AZ	2,082	609.6	43,608	66	CGAAGGGGCAT	69

Genome extraction was performed from high-titer lysates using the Wizard DNA cleanup kit (Promega), and sequencing was performed at the University of Pittsburgh. Libraries were constructed using the NEBNext Ultra II FS DNA library preparation kit and sequenced using an Illumina MiSeq v3 platform generating 150-bp unpaired ends. Raw reads were assembled using Newbler v2.9 with default settings, generating single contigs with coverage of approximately 2,651× for Phives, 2,171× for Kaylissa, and 2,081× for Eraser (7). The phage contigs were checked using Consed v29 to evaluate completeness and determine genomic termini (8). The genome parameters (length, GC content, and termini) and accession numbers (GenBank and SRA) are shown in Table 1.

All bioinformatic analyses and software were used with default parameters. The three phages were assigned to cluster AZ based on shared gene content similarity (GCS) exceeding at least 35% using the online tool at the PhagesDB database (https://phagesdb.org/genecontent/) (9, 10). Coding regions were predicted using GeneMark v3.25 (11) and GLIMMER v3.02b (12) and subsequently manually curated using DNA Master v5.23.6 (13), Phamerator (14), BLAST (15), and Starterator v1.0.1 and v1.2 (http://phages.wustl.edu/starterator). No tRNA genes were identified with ARAGORN v1.2.41 (16). Functions for each coding sequence were evaluated using NCBI BLASTP v2.9 (15), HHpred v3.2 (17), and Phamerator (14). Membrane proteins were predicted using TMHMM v2.0 (18).

Phives, Kaylissa, and Eraser have genome architectures consistent with *Arthrobacter* phage cluster AZ genomes. The genomes of all three phages have defined ends with 11-base, complementary, 3′ single-stranded extensions. Predicted genes in the left halves of the genomes are well conserved among the three phages and code for virion structure and assembly proteins. The right halves of the genomes are less well conserved, and predicted genes with no known function are prevalent. Phives, Kaylissa, and Eraser contain a serine integrase gene and are predicted to be temperate, although no repressor gene has been identified. The putative endolysin genes for these phages are in different locations within the genomes (Phives, gene 1; Eraser, gene 25; Kaylissa, gene 59), and they share less than 31% amino acid identity with each other.

### Data availability.

GenBank and Sequence Read Archive (SRA) accession numbers for phages Phives, Kaylissa, and Eraser are provided in Table 1.
